# Supplementary material for: Enhancing performance of subject-specific models via subject-independent information for SSVEP-based BCIs
Source: PLoS One. 2020 Jan 14;15(1):e0226048. doi: 10.1371/journal.pone.0226048 (PMC6959579; doi:10.1371/journal.pone.0226048)
Supplement: S1 Appendix — (DOCX) [file pone.0226048.s001.docx]

**S1 Appendix: Feature selection results**

As described in section 2.4.1, a forward selection (FS) approach was used to find the best correlation coefficients derived from CCA-based spatial filters among 36 correlation features (Fig 2). This procedure was repeated for each fold using a subject-independent training schema. Feature selection was done with 0.5 s data length. Surprisingly, for each fold, the same set of features was found, and the maximum accuracy was obtained using the best six features. The results of each fold for these features are shown in the Table S1. CVs are the canonical variables defined in Table 1, and the numbers inside the parentheses show classification accuracy for each stage of the selection process. Note that for computing the accuracy of the *k-*th fold (*k-*th row), the subjects of that fold were left out, and the classification accuracy was computed using the remaining 30 subjects.

As can be seen, although the order of selection is different for each fold, the same set of features is obtained. Cells with a blue background represent features introduced in this paper and cells with white background represent features used previously in an extended CCA method [12]. The correlation coefficient $\rho(\mathrm{CV}_{7},\mathrm{CV}_{10})$ obtained by the proposed method is the best feature in four folds. The logic behind selection of these features is clearly described in the proposed method.

S1 Table. Selection of best six features for seven folds. The numbers (inside the parentheses) in the *n-*th column indicate classification accuracy with the best *n* features.

| 6^th^ coeff | 5^th^ coeff | 4^th^ coeff | 3^rd^ coeff | 2^nd^ coeff | 1^st^ coeff^*^ |  |
| --- | --- | --- | --- | --- | --- | --- |
| $\rho(\mathrm{CV}_{5},\mathrm{CV}_{9})$  (63 %) | $\rho(\mathrm{CV}_{1},\mathrm{CV}_{4})$  (62.4 %) | $\rho(\mathrm{CV}_{7},\mathrm{CV}_{10})$  (60.8 %) | $\rho(\mathrm{CV}_{1},\mathrm{CV}_{2})$  (58.9 %) | $\rho(\mathrm{CV}_{5},\mathrm{CV}_{6})$  (57.8 %) | $\rho(\mathrm{CV}_{7},\mathrm{CV}_{8})$  (51.8 %) | fold 1 |
| $\rho(\mathrm{CV}_{1},\mathrm{CV}_{4})$  (67 %) | $\rho(\mathrm{CV}_{7},\mathrm{CV}_{8})$  (66.7 %) | $\rho(\mathrm{CV}_{5},\mathrm{CV}_{9})$  (64.5 %) | $\rho(\mathrm{CV}_{5},\mathrm{CV}_{6})$  (63.8 %) | $\rho(\mathrm{CV}_{1},\mathrm{CV}_{2})$  (62.1 %) | $\rho(\mathrm{CV}_{7},\mathrm{CV}_{10})$  (55.1 %) | fold 2 |
| $\rho(\mathrm{CV}_{1},\mathrm{CV}_{4})$  (65.8 %) | $\rho(\mathrm{CV}_{7},\mathrm{CV}_{10})$  (65.1 %) | $\rho(\mathrm{CV}_{5},\mathrm{CV}_{9})$  (64.2 %) | $\rho(\mathrm{CV}_{5},\mathrm{CV}_{6})$  (62.5 %) | $\rho(\mathrm{CV}_{1},\mathrm{CV}_{2})$  (61 %) | $\rho(\mathrm{CV}_{7},\mathrm{CV}_{8})$  (54.2 %) | fold 3 |
| $\rho(\mathrm{CV}_{1},\mathrm{CV}_{4})$  (67.8 %) | $\rho(\mathrm{CV}_{7},\mathrm{CV}_{10})$  (67 %) | $\rho(\mathrm{CV}_{5},\mathrm{CV}_{9})$  (66.1 %) | $\rho(\mathrm{CV}_{5},\mathrm{CV}_{6})$  (64.3 %) | $\rho(\mathrm{CV}_{1},\mathrm{CV}_{2})$  (62.1 %) | $\rho(\mathrm{CV}_{7},\mathrm{CV}_{8})$  (56.2 %) | fold 4 |
| $\rho(\mathrm{CV}_{1},\mathrm{CV}_{4})$  (64.6 %) | $\rho(\mathrm{CV}_{7},\mathrm{CV}_{8})$  (64 %) | $\rho(\mathrm{CV}_{5},\mathrm{CV}_{9})$  (63.3 %) | $\rho(\mathrm{CV}_{5},\mathrm{CV}_{6})$  (62.2 %) | $\rho(\mathrm{CV}_{1},\mathrm{CV}_{2})$  (60 %) | $\rho(\mathrm{CV}_{7},\mathrm{CV}_{10})$  (52.7 %) | fold 5 |
| $\rho(\mathrm{CV}_{1},\mathrm{CV}_{4})$  (64.7 %) | $\rho(\mathrm{CV}_{7},\mathrm{CV}_{8})$  (64 %) | $\rho(\mathrm{CV}_{5},\mathrm{CV}_{9})$  (63.2 %) | $\rho(\mathrm{CV}_{5},\mathrm{CV}_{6})$  (62 %) | $\rho(\mathrm{CV}_{1},\mathrm{CV}_{2})$  (59.1 %) | $\rho(\mathrm{CV}_{7},\mathrm{CV}_{10})$  (53 %) | fold 6 |
| $\rho(\mathrm{CV}_{1},\mathrm{CV}_{4})$  (65.2 %) | $\rho(\mathrm{CV}_{7},\mathrm{CV}_{8})$  (64.6 %) | $\rho(\mathrm{CV}_{5},\mathrm{CV}_{9})$  (63.8 %) | $\rho(\mathrm{CV}_{5},\mathrm{CV}_{6})$  (61.8 %) | $\rho(\mathrm{CV}_{1},\mathrm{CV}_{2})$  (59.5 %) | $\rho(\mathrm{CV}_{7},\mathrm{CV}_{10})$  (53.4 %) | fold 7 |

^*^coeff= coefficient
