# Supplementary material for: Enhancing performance of subject-specific models via subject-independent information for SSVEP-based BCIs
Source: PLoS One. 2020 Jan 14;15(1):e0226048. doi: 10.1371/journal.pone.0226048 (PMC6959579; doi:10.1371/journal.pone.0226048)
Supplement: Attachment — Submitted filename: Response.to.Reviewers.docx [file pone.0226048.s002.docx]

**Point-by-Point Responses to the Reviewers’ Comments**

We thank the reviewers for their valuable comments and suggestions, based on which we have revised the manuscript. We have kept the track of the changes in the revised manuscript and provided our point-by-point responses to the comments below.

**Reviewer 1**

**Comment 1:** Authors used publicly available 35-subject SSVEP benchmark dataset to evaluate the proposed method, and data of 30 subjects is utilized to obtain the best hyperparameters for the remaining 5 subjects for cross-validation, is the number of subjects enough for comparation?

**Response:** The mentioned process was repeated 7 times for the 7 folds. For each fold, the data of 30 subjects were used to find the best hyperparameters. Then, these hyperparameters were used for the remaining 5 subjects. Therefore, we used the data of all 35 subjects (7 times 5) in the evaluation process. We concatenated the results of all folds (the output became a vector with a length of 35) and averaged across the subjects. Therefore, the reported results are the average performance across all subjects. The number of subjects is sufficient for the comparison, similar to previous works that used this dataset.

**Comment 2:** The figures are arranged in all of the paper including methods, results and discussion, it seems a little messy. In addition, authors may consider expanding the heading fonts of abscissa and ordinate, such as Figure 3 and Figure 4.

**Response:** Majority of the figures (6 out of 8) are presented in the Methods and Results Sections to explain the proposed methods and the results obtained from their applications. We used two figures in the Discussion Section to illustrate and compare additional features of the methods. We have applied the suggested changes to Figures 3 and 4.

**Comment 3:** Authors may pay attention to the format of references, such as reference 18 and 39.

**Response:** We have corrected the format of references including references 18 and 39 in the revised manuscript.

**Reviewer 2**

**Comment 1:** The figures are not clear. Please provide high resolution figures.

**Response:** We think the problem is with the pdf file generated by the online submission website. As acknowledged by PLOS ONE, the quality of the images in the generated pdf may be low. However, PLOS ONE provides a link for dowloading the high-quality version of each image. Our submitted figures had a resolution of 300 dpi and the dimensions were corrected using <https://pacev2.apexcovantage.com> as required by the journal. To be on the safe side, we double-checked our submitted images on the PLOS ONE website and confirmed their high quality. Please let us know if there is any additional requirements (e.g., a resolution higher than 300 dpi or a dimension larger than the current version). Also, the .mat version of the images is available if needed.

**Comment 2:** The paper is written in poor English. It requires extensive language revision, possibly by a native English speaker.

**Response:** We have extensively revised the manuscript to improve its English.

**Comment 3:** Please describe the broad value and interest of your new approach.

**Response:** The advantages of our approach relative to the TRCA and extended CCA methods (two state-of-the-art methods) for target detection in SSVEP-based BCI are the following, which are also presented in the revised manuscript.

- Our method integrates subject-specific models with subject-independent information and enhances the BCI performance.
- The classification accuracy and information transfer rate (ITR) of our method are significantly higher than those of the extended CCA in all conditions and those of TRCA in time windows larger than 0.3 s.
- Our method can be easily implemented in online applications of BCI and realize a high-speed SSVEP based speller.
- Our method outperforms TRCA when the number of the training blocks and the number of the electrodes are small. Also, for subject-specific training, TRCA needs at least two training blocks while our method works with a single training block. This facilitates the development and application of the BCI systems.
- A problem with the SSVEP-based BCI spellers is false detection, due to interference from the nearest neighbors of the target frequency. The likelihood of this error for our method is lower than that of the TRCA method.
